# Costing evidence for health care decision-making in Austria: A systematic review

**DOI:** 10.1371/journal.pone.0183116

**Published:** 2017-08-14

**Authors:** Susanne Mayer, Noemi Kiss, Agata Łaszewska, Judit Simon

**Affiliations:** 1 Department of Health Economics, Center for Public Health, Medical University of Vienna, Vienna, Austria; 2 Ludwig Boltzmann Institute Applied Diagnostics, General Hospital Vienna, Vienna, Austria; University of Washington Department of Global Health, UNITED STATES

## Abstract

**Background:**

With rising healthcare costs comes an increasing demand for evidence-informed resource allocation using economic evaluations worldwide. Furthermore, standardization of costing and reporting methods both at international and national levels are imperative to make economic evaluations a valid tool for decision-making. The aim of this review is to assess the availability and consistency of costing evidence that could be used for decision-making in Austria. It describes systematically the current economic evaluation and costing studies landscape focusing on the applied costing methods and their reporting standards. Findings are discussed in terms of their likely impacts on evidence-based decision-making and potential suggestions for areas of development.

**Methods:**

A systematic literature review of English and German language peer-reviewed as well as grey literature (2004–2015) was conducted to identify Austrian economic analyses. The databases MEDLINE, EMBASE, SSCI, EconLit, NHS EED and Scopus were searched. Publication and study characteristics, costing methods, reporting standards and valuation sources were systematically synthesised and assessed.

**Results:**

A total of 93 studies were included. 87% were journal articles, 13% were reports. 41% of all studies were full economic evaluations, mostly cost-effectiveness analyses. Based on relevant standards the most commonly observed limitations were that 60% of the studies did not clearly state an analytical perspective, 25% of the studies did not provide the year of costing, 27% did not comprehensively list all valuation sources, and 38% did not report all applied unit costs.

**Conclusion:**

There are substantial inconsistencies in the costing methods and reporting standards in economic analyses in Austria, which may contribute to a low acceptance and lack of interest in economic evaluation-informed decision making. To improve comparability and quality of future studies, national costing guidelines should be updated with more specific methodological guidance and a national reference cost library should be set up to allow harmonisation of valuation methods.

## Introduction

To assess the overall value of a health intervention, decision makers need information on the effect, the resources used to generate the effect, and the unit cost of these resources. Inaccuracies in any of these three pieces of information increase the risk of incorrect inferences including potentially favouring health interventions or policies that leave society worse off than their alternatives, or rejecting those that could provide additional benefit to the society. Internationally, the methodology for measuring the effects of an intervention is a well-standardised process, whereas methods for costing processes have received comparatively little attention [1]. Practical guidance on costing methods is lacking [2–5]. Although it is internationally agreed that resource use is to be measured in the smallest unit necessary for comparison purposes within or between economic analyses(s) (e.g. one hour of physiotherapy, or one dose of a certain medication) [6, 7], the availability of standardised and validated resource use measurement tools is limited [8, 9]. In addition, the methods of valuing measured resources in terms of their unit costs are not internationally harmonised and remain context specific [2].

From an economic perspective, valuation of resource use should be based on opportunity costs, i.e. the forgone value as the resources are no more available for their next best alternative use [6], sometimes referred to as ‘economic cost’ [10]. In a perfectly competitive market, this opportunity cost is reflected in the market price. However, due to the heavy influence of governmental regulation and stakeholder negotiations, health care is typically considered as functioning imperfectly. Existing market prices (e.g. reimbursement data, tariff catalogues and other administrative sources) are nevertheless commonly used for valuation [10]. However, these prices do not necessarily capture the ‘true value' of the resource forgone [11].

Different health care systems have established varying degrees of standardisation of costing methods at national and/or regional levels [2]. This heterogeneity weakens economic analyses, impeding not only the comparison, interpretation and transferability of the cost analyses, but also resulting in methodological criticism in general [4]. Coincident with the publication of unclear analyses, this lack of standardization further limits the scope and utilisation of such evidence in decision-making nationally and internationally.

The Austrian health care system is a Bismarck-type, dominantly social insurance-based health care system, and provides coverage for 99.9% of the Austrian population [12, 13]. With health care spending amounting to 11.1% of gross domestic product (GDP) in 2015 [14], Austrian health care expenditures are above the OECD average. In comparison to other OECD countries, a relatively higher share of spending is invested e.g. in inpatient care and a lower share in outpatient care [15]. Hospital care provided by public and non-profit hospitals is reimbursed using an Austrian version of the Diagnosis-Related Groups-based (DRG) (Austrian LKF system) payment system [13]. A total of 19 statutory health insurance funds under the umbrella of the Main Association of Social Security Institutions are responsible for financing practice-based ambulatory care. Out of the 19 health insurance funds, the nine regional health insurance funds cover around 75% of the Austrian population, and the four occupational and six company insurance funds cover the remaining 25% [13]. Services provided by physicians in a contractual relationship with one or more statutory health insurance funds are reimbursed based on health insurance fund specific tariff catalogues applying a mixed payment system [13]. These health care system characteristics result in a high level of fragmentation at funding, planning and delivery levels [13], and have major implications in terms of how the role of economic evaluations is currently seen and considered in relevant national/regional decision-making processes.

In Austria, economic evaluations were introduced as a fourth hurdle (in addition to efficacy, safety and quality) in the context of reimbursement decisions for innovative pharmaceuticals in the outpatient sector in 2002 [16]. In this process, cost-effectiveness is used as a formal criterion, but no threshold is applied for decision-making [17] and reimbursement decision reports are not publicly available [18]. Economic evaluation plays a minor role in determining reimbursement for other products and services [19]. In 2006, the first Austrian guideline for economic evaluations was published [19], albeit with minimal information on costing methods. In the past decade, a growing number of economic evaluations have been published, partly also attributable to the formation of a Ludwig Boltzmann Institute for Health Technology Assessment in 2006. Additionally, the importance of economic evaluations is to be expected to increase in the future due to the growing role of Health Technology Assessments (HTA) in the last Austrian health care reform [20] and at an international level [21], as well as due to the establishment of relevant professional associations such as the Austrian Health Economics Association (ATHEA) [22] and the ISPOR Austrian Chapter [23] in the past five years. Awareness of the necessity to use national cost data to produce relevant evidence and assist national-level decision-making has also grown [24]. To the present day, however, national-level unit cost data have not been systematically collected and/or have been made publicly available across relevant sectors in Austria [19].

In light of this institutional background, this review aims to systematically synthesise and appraise published health economic analyses and their relevant costing methods since the initiation of the Austrian economic evaluation guidelines [19]. Specifically, this methodological review examines the studies’ valuation approaches and unit cost sources in detail and thus maps the current costing landscape together with future development opportunities in this area [25]. In addition, the practical use of the information collected in this review is intended as a point of reference for available cost sources and values for future economic evaluations in an Austrian setting. It aims to promote consistency and quality in cost reporting to improve the usability of future economic evaluations in decision-making and give context to the updating of national costing guidelines and the establishment of a national reference cost library.

## Methods

A systematic literature review was conducted on the peer-reviewed as well as grey literature in the Austrian context published between 2004 and 2015 in English or German language. The methodology applied is based on the Guideline for Conducting Systematic Literature Reviews in Economic Evaluation provided by the Centre for Reviews and Dissemination, University of York [26]. The following electronic databases were searched in December 2015: MEDLINE, EMBASE, Scopus, Social Science Citation Index (SSCI), EconLit, and the NHS Economic Evaluation Database (NHS EED). No review protocol exists. Search terms (S1 Text) were based on SIGN search filters for economic studies. MeSH and EMTREE terms were used where appropriate.

Two levels of screening were conducted for each reference: 1) title and abstract, and 2) full text. Each study was assessed independently by two reviewers (NK, AL) at both stages. Discrepancies were mitigated by a third reviewer (SM). Studies were included if they were full text (not abstracts or posters) health economic analyses published between 2004 and 2015 that used cost data pertaining to Austria. Studies were considered if they contained full economic evaluations that used evidence on both the effects and costs when comparing two or more alternative interventions, partial economic evaluations focusing only on cost comparisons (cost analyses), as well as cost descriptions (e.g. cost-of-illness studies, budget impact analyses) [1]. For the grey literature search, key institutions were identified based on the Austrian HTA guide [27] (S2 Text). Where the full text of a publication was not available, the corresponding author was contacted. An extraction table was developed jointly by the project team and piloted by the three reviewers (SM, NK, AL). The final extraction table included the following items: study details (authors, year, title, journal, article impact factor, language, conflict of interest, funding body), features of the study design (study type, disease area, level of care and intervention, study perspective), information on costs (type of costs, geographical area of costs, cost categories, sources of costs by sector, stated limitations of cost (sources), adjustments to costs, year of costs). Each included study was extracted and double checked by a second reviewer for accuracy.

The quality of costing methods in the studies was evaluated by comparing extracted information on the reporting of unit costs and their sources based on the Austrian guidelines for economic evaluations [19] and combined relevant items from international checklists for economic evaluation [26, 28, 29]. Specifically, three quality aspects were assessed: Firstly, we assessed whether or not the sources used or calculation methods applied to obtain or value the unit costs were clearly reported. Secondly, we assessed if the ‘ingredients approach’ [30] was followed, i.e. if the unit costs were reported separately from the resource quantities. And thirdly, we assessed whether limitations in the context of using specific sources for the valuation of resource use data were discussed by the study authors.

## Results

**Fig 1:**
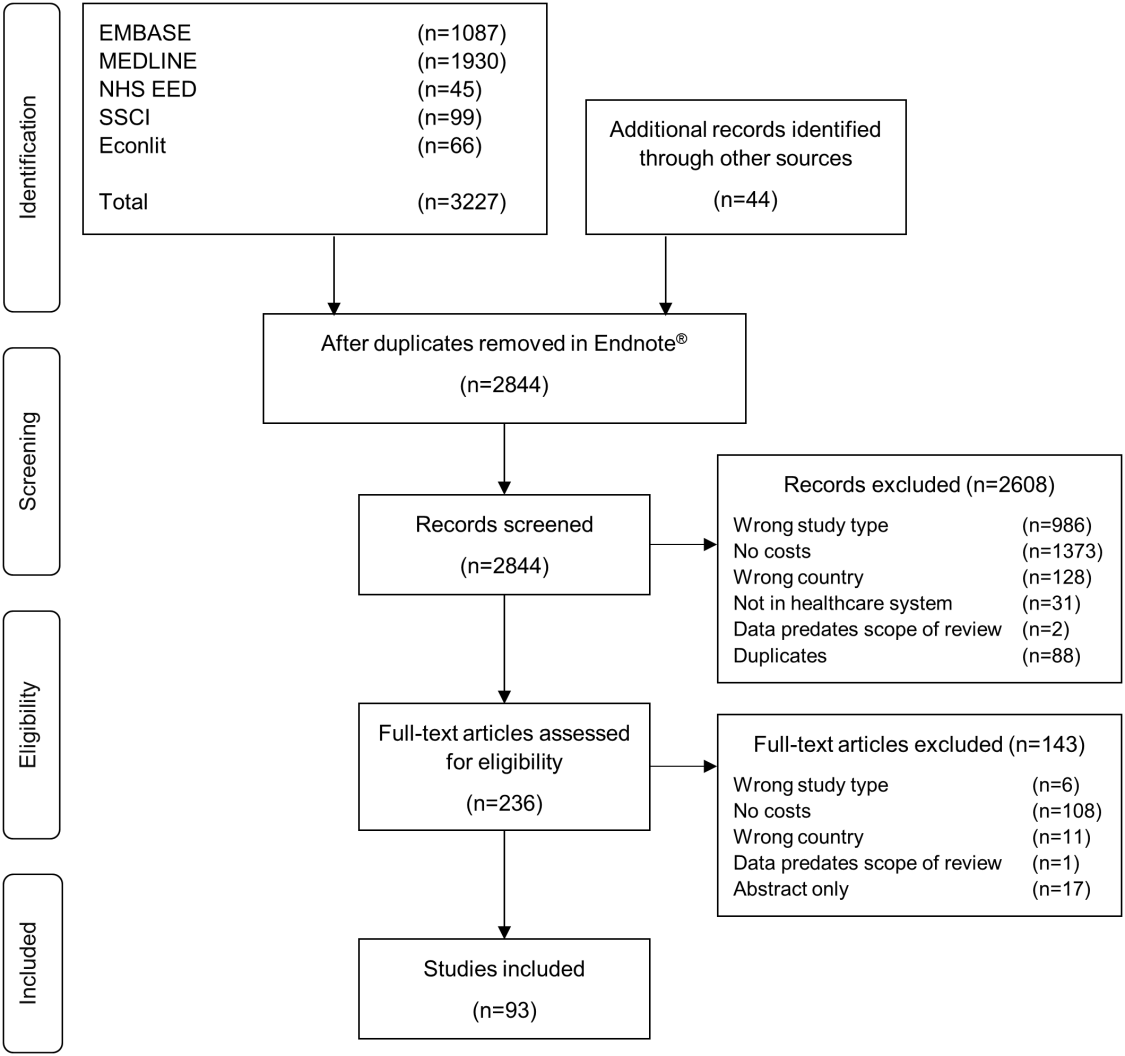
PRISMA. PRISMA flow diagram.

Fig 1 shows the flow of studies identified, screened, and included in the review in the form of a PRISMA chart [31]. The search initially identified 2,844 studies after deduplication. A total of 93 studies fulfilled all inclusion criteria, were extracted and analysed.

### Publication characteristics

**Fig 2:**
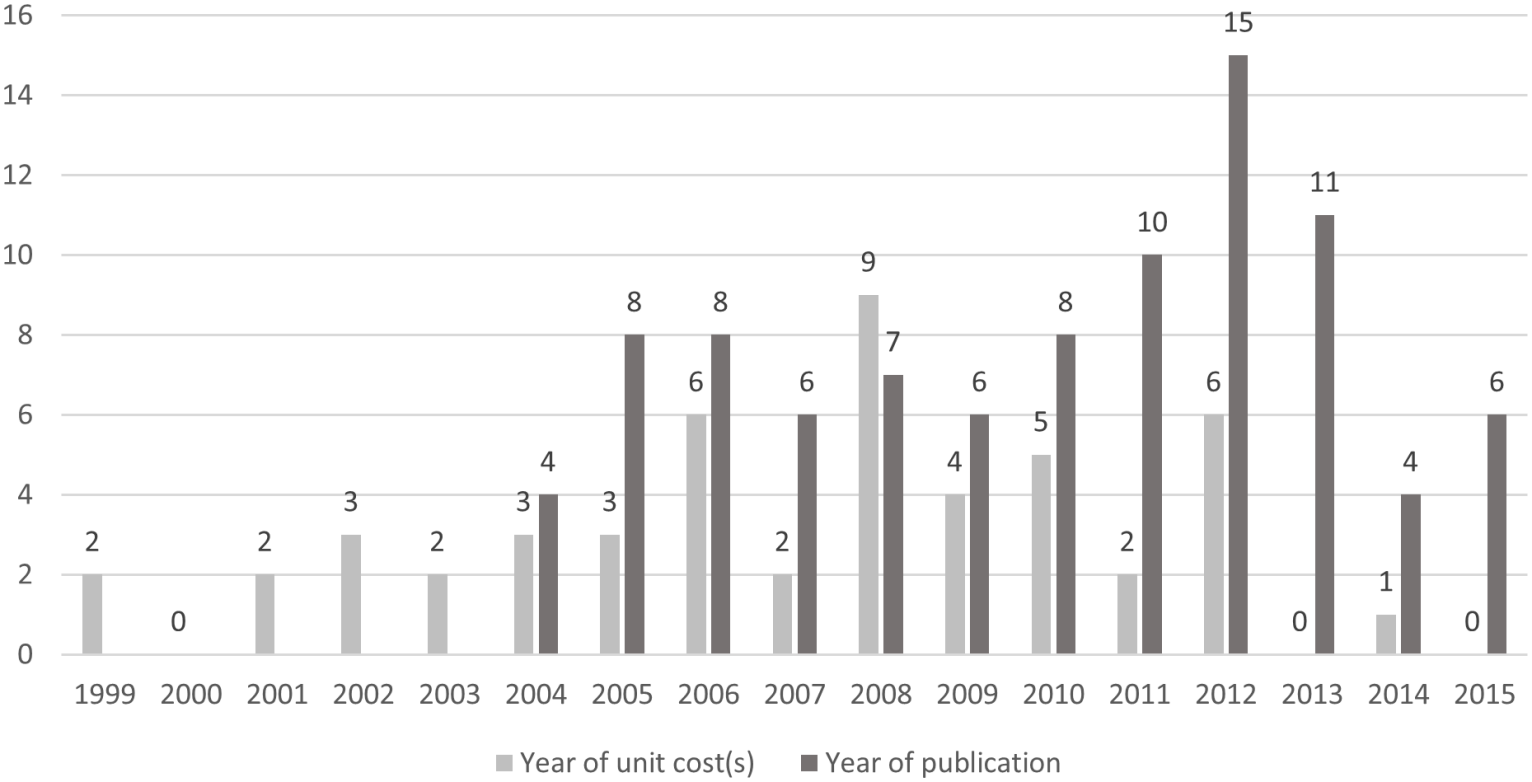
Year of publications and unit costs. Number of publications and year of reported unit costs, by year.

The publication characteristics of the included studies are presented in Table 1. The largest number of studies was published in 2012 (n = 15) and 2013 (n = 11), with an average of 7.8 studies published annually (Fig 2). The majority of them (n = 81, 87%) were journal articles, the remaining studies were reports (n = 12, 13%). While journal articles were mostly published in English (61 out of 81, 75%), reports were typically in German language (ten out of 12, 83%). Of all journal articles, 72% (58 out of 81) were published in a journal indexed in JCR (Journal Citation Reports) with an average impact factor of 2.2 in the year of publication (English articles: 2.3; German articles: 0.85). Based on subject categories, only a minority of these indexed articles (15 out of 58, 26%) were published in health economics, public health or health services journals; the majority (43 out of 58, 74%) were published in clinical medicine journals. Fifty-two studies (56%) explicitly declared information on potential sponsorship, with nine (10%) specifically reporting no external funding. Among the 43 studies with explicitly reported funding sources (46%), four received funding from multiple sources and 39 from single sources. Information on relevant funding bodies are listed in Table 1. In half of the studies (n = 46, 50%) at least one international institution was involved in the publication, mostly located in Germany (n = 32), in the USA (n = 20), in Switzerland (n = 17) and in the UK (n = 17). Twenty-one studies (23%) did not exclusively focus their analysis on Austria, but were multi-country analyses and included other countries in Europe (n = 19) or in North America (n = 2).

**Table 1:** Publication characteristics (n = 93).

	No. of studies	%
**Type of publication**		
Journal article	81	87
JCR indexed*	58	72
Health economics, public health, health services	15	26
Clinical medicine	43	74
Non-JCR indexed	23	28
Report	12	13
**Publication language**		
English	63	68
German	30	32
**Disclosure of funding source(s)**		
Disclosed: Funding body stated^†^	43	46
No external funding	9	10
Not disclosed	41	44
**Geographical region covered in economic evaluation**		
Austria only	72	77
Multi-national	21	23

Note:

*Journals indexed according to Journal Citation Reports^®^ (Social) Sciences Edition. Five most common journals: Wiener klinische Wochenschrift*, Wiener Medizinische Wochenschrift, Pharmacoeconomics*, Expert Review of Pharmacoeconomics & Outcomes Research, Journal of Medical Economics.

^†^ Five most common funding sources: industry (pharma companies, medical device companies, Austrian ministries and thereby funded grant bodies, international funding bodies (mostly European Union), health insurance funds.

### Study characteristics

The basic study characteristics are summarised in Table 2. Thirty-eight studies (41%) were full economic evaluations, of which 26 studies were model-based. Among the other 55 studies (59%), 18 studies were comparative cost analyses and 37 were cost descriptions, and only four studies were model based. By ICD-10 subject areas (International Statistical Classification of Diseases and Related Health Problems, 10^th^ Revision, [32]), most studies fell into chapter ‘IX Diseases of the circulatory system’ (n = 15, 16%), followed by chapter ‘XXI Factors influencing health status and contact with health services’ (n = 12, 13%) and chapter ‘II Neoplasms’ (n = 11, 12%). By level of care, the majority of studies dealt with curative interventions (n = 58, 63%).

**Table 2:** General study characteristics (n = 93).

	No. of studies	%
**Type of study**		
Full economic evaluation	38	41
Cost-minimization analysis	3	3
Cost-effectiveness analysis	25	27
Cost-utility analysis	3	3
Cost-effectiveness and cost-utility analysis	7	8
Cost analysis	18	19
Cost description	37	40
Budget impact	3	3
Cost-of-illness	14	15
Others (e.g. cost of treatment)	20	22
**Model-based study**	30	33
**ICD-10 subject area**		
IX Diseases of the circulatory system	15	16
XXI Factors influencing health status and contact with health services	12	13
II Neoplasms	11	12
XIII Diseases of the musculoskeletal system and connective tissue	8	9
I Certain infectious and parasitic diseases	8	9
IV Endocrine, nutritional and metabolic diseases	7	7
V Mental, behavioural and neurodevelopmental disorders	7	7
Other ICD-10 chapters	25	27
**Level of care and intervention type**		
Primary prevention	7	7
Secondary prevention	23	25
Tertiary prevention	5	5
Curative (surgical/medical procedures)	35	38
Curative (pharmaceuticals)	23	25

### Costing methods and reporting

Details of the costing methods and reporting standards are summarised in Table 3. Table 3 shows that 37 of all studies (40%) explicitly reported the study perspective(s), with the most commonly stated perspective being that of the payer (n = 26, 28%) followed by the societal perspective (n = 14, 15%). Eleven studies (12%) reported results from more than one perspective. A significantly higher proportion of full economic evaluations (21 out of 38, 55%) was explicit about this aspect than of the other types of studies (16 out of 55, 29%).

**Table 3:** Costing methods (n = 93).

	No. of studies	%
Study perspective*		
Not stated	56	60
Payer	26	28
Provider	6	6
Patient	2	2
Societal	14	15
**Included cost components**		
Health and social care sector		
Inpatient	70	75
Hospital outpatient/day patient	32	34
Physician practice	33	35
Medication	46	49
Other health care (e.g. medical devices)	26	28
Rehabilitation	3	3
Long-term care	8	9
Other social care (e.g. social worker)	4	4
Patient/family costs		
Patient/waiting time	1	1
Travel expenses	3	3
Informal care	5	5
Other patient costs (e.g. prescription fee)	4	4
Productivity losses	24	26
Criminal justice sector	1	1
**Level of study costing**		
National	55	59
Regional	38	41
**Year of applied unit cost(s)**		
Clearly stated	70	75
Adjustments to unit costs*		
Inflating	17	18
Discounting	18	19
Other (e.g. purchasing power adjustment)	4	4
Not stated	61	68
**Reporting of applied unit costs**		
Complete	58	62
Partial	35	38
**Reporting of applied unit cost sources**		
Complete	68	73
Partial	25	27
**Application of micro-costing methods**	15	16

Note:

*Since some studies applied more than one study perspective, values do not add up to 100%.

Following the cost categorisation by Drummond and colleagues [33], inpatient costs (n = 70, 75%), outpatient costs (hospital-based: n = 32, 34%; physician practice-based: n = 33, 35%) and medication costs (n = 46, 49%) were the most frequently included health and social care cost components. Costs from other sectors referred to patient and family costs in the form of informal caregiving in five studies (5%) and the criminal justice sector in one study (1%). Around one in four studies (n = 24, 26%) incorporated lost productivity, in line with the Austrian guidelines applying the method of human capital approach [19] if reported. In the majority of the studies (n = 55, 59%), cost analyses were used for national-level inferences, the others (n = 38, 41%) used regional-level or specific local provider-level inferences, often irrespective of the generalisability of the applied unit costs. The year of costs was stated by 75% (n = 70) of all studies and ranged from 1999 to 2014. Overall, 18% (n = 17) of all studies inflated the costs to the year of their analysis, with one study using a health-specific inflation rate [34]. This adjustment was justified in all instances (n = 17). By contrast, a comparison of how many other studies should have adjusted their costs but did not is not feasible in light of the limited details provided in some studies regarding e.g. the year of cost or missing additional information. In 19% (n = 18) of all studies, future costs (and in some cases: outcomes) were discounted, typically applying a discount rate of 2–5% in their main analysis (5% recommended in the Austrian guidelines [19]). There was an average time lag of three years between the base year of costing and publication of the study (Fig 2).

Seventeen studies (18%) referenced economic evaluation guidelines in connection with their adopted costing methodology. In around half of them (eight out of 17, 47%) the authors referred to the Austrian guidelines for economic evaluation from 2006 [19] or the Austrian HTA methods handbook from 2012 [24], followed by guidelines from Germany (n = 4, 24%) or other countries (n = 5, 29%). While 62% of all studies (n = 58) comprehensively reported all unit costs separately from the respective resource use information, all unit cost sources (or if applicable: calculation methods) were comprehensively listed in 73% (n = 68) of all studies. Hence in several studies, unit cost sources were missing but sources listed.

**Fig 3:**
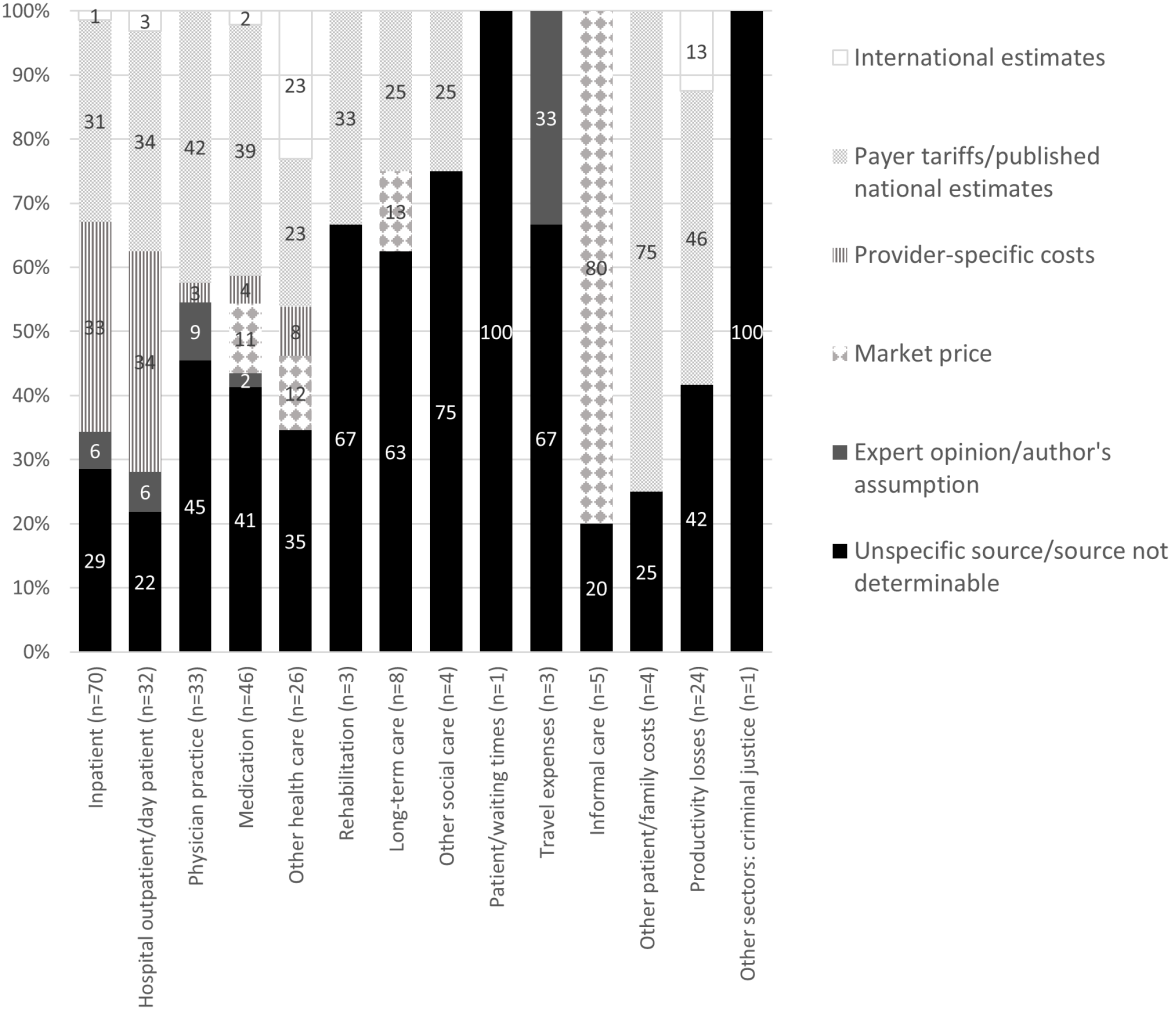
Sources of costs. Valuation of costs, by sector.

Regarding the origin of the unit costs, a variety of sources were identified, often also in the case of the same cost component (Fig 3). For example, the unit costs for a general practitioner (GP) consultation were found to be taken from a variety of sources including tariffs from single regional health insurance funds, a weighted average tariff from more than one regional health insurance fund, internal estimate of the Main Association of Social Security Institutions, expert advice or non-specified sources. In line with the heterogeneity of the listed sources, also the unit costs for a GP visit varied considerably. A difference of approximately 135% could be observed between the lowest and highest reported unit cost for a (non-disease specific) GP contact (all values inflated to year 2015) (n = 7). The vast impact of the source of valuation and costing methodology on the derived estimates is supported by another cost analysis identified in this review [35]. When comparing the costs of day surgery for varix operation based on different data sources and/or methods (including tariff-based costing, provider specific micro-costing and international cost data), the difference in the unit cost estimates amounted to around 450% [35].

Depending on the specific cost component considered in the reviewed studies (Fig 3), a varying number of studies relied on expert opinion (which is an acknowledged source according to the Austrian guidelines [19]) and/or author assumptions. These sources were used most commonly for travel costs, services in physician practices and the hospital sector. The number of studies listing only unspecific sources of valuation or no determinable source ranged from 20% (informal care) to 100% (criminal justice sector, patient/waiting times) depending on the cost component. As opposed to hospital outpatient and inpatient services, sources for unit costs in physician practices were typically not comprehensively reported. Austria-specific unit costs for the hospital sector came mostly from provider-specific sources (i.e. institution-based accounting information) (n = 23, 33%) or payer tariffs (i.e. centrally determined reimbursement values including diagnosis-related groups (DRG) data) (n = 22, 31%). For resource use in physician practices (n = 14, 42%) and medication (n = 18, 39%), payer tariffs were the main source of valuation, while for medication also market prices were utilised (n = 5, 11%). Overall, ten studies (11%) relied on unit costs from international sources, including unit costs from Germany, the UK, and Sweden, which were mostly used for other medical services (e.g. specific diagnostic tests, surgical procedures).

The application of micro-costing methods was also examined. According to Gold and colleagues [36], micro-costing (also known as ‘bottom-up’ costing or ‘activity-based costing’ [37]) involves the ‘direct enumeration and costing out of every input consumed in the treatment of a particular patient’ and is considered the ‘gold standard’ in economic evaluation [38]. Overall, 15 studies (16%) employed this approach to value resource use for all (ten out of 15, 67%) or some resource use items (five out of 15, 33%) included in their analysis [39–53]. Micro-costing was mostly applied when dealing with the hospital sector, except for one study [45] that used the method to value an ambulatory care service.

Finally, we assessed if limitations arising in the context of the valuation of resources were discussed in the studies. Such discussion is especially relevant when e.g. tariffs are used as a substitute for the opportunity cost of a service (Fig 3). The necessity to use payer tariffs due to the lack of available ‘true’ economic costs was the most commonly acknowledged limitation in the reviewed publications [54–59] (n = 6, 6%). One of these studies also explicitly pointed out that such proxy cost data have been accepted as standard source of valuation in relevant health economics textbooks [54].

## Discussion

This is the first review synthesising key characteristics and costing methods of published health economic analyses in Austria. It is also the first analysis looking into these details in the context of Central and Eastern European (CEE) healthcare systems where this area of evidence generation is still lacking behind in numerous international comparisons. In Austria, an average of 7.8 relevant studies (Fig 1) have been published annually since 2004. This is a relatively low number when compared to other German speaking countries. For example, in Germany an average of 18.9 full economic evaluations were published annually between 1990 and 2004 [60]. The h-index of health economics publications was found to be more than 2.8 times higher in Germany and more than 2.5 times higher in Switzerland than in Austria [61]. These data support the notion that both health economics and more narrowly, economic evaluations, are still evolving research and decision support tools also in Austria. One of the possible explanations commonly brought up by decision-makers in this context is the lack of quality and relevance of costing to the Austrian setting [62]. The aim of this review was to map the relevant Austrian economic analysis landscape with a special focus on synthesising the applied costing methods and reporting standards in order to identify the potential relevant main hurdles in acceptance and areas for improvement.

The study perspective of an economic analysis is one of the pivotal parameters affecting the choice of costs to be included. It also determines which sources may be relevant/acceptable for valuation. While e.g. the available tariffs are appropriate cost sources from a payer perspective, they do not necessarily reflect economic costs from the provider’s viewpoint. Only 40% of the identified studies explicitly stated the study perspective and most did not include patient and family costs (e.g. travel costs) or inter-sectoral costs (e.g. based on resource use in the criminal justice or education sector) contributing to a fully societal perspective [63]. This finding is in line with an earlier international review of cost-utility analyses showing a general difficulty of obtaining relevant resource use and unit cost information across several countries [64]. The applied costing methods summarised in Table 3 and Fig 3 reflect also the availability and easiness of access to certain data types and sources in Austria with information on inpatient, medication and physician practice tariffs and other reimbursement data being the most readily available.

The quality assessment of the reporting standards revealed several further problem areas. Firstly, insufficient reporting of the year of the unit cost as seen in 25% of all studies (contrasting e.g. the 90% of reviewed studies in Saudi Arabia, [65]) in this review impairs the reproducibility and transferability of the results, or the opportunities for necessary updates due to technological advances. Secondly, even though references to the source of all applied unit costs were given by 73% of the studies, which is comparable to other countries [66], this information in many cases was not described in sufficient detail to allow the tracking down of the actual source. For example, quite commonly only unspecific sources of valuation were provided for the inpatient sector with reference to the “LKF” (short for Austrian DRG system), for practice-based physicians in the form of “tariffs”, and for medication referencing “sickness fund prices” (‘Kassenpreise’). Thirdly, only 62% of the reviewed studies followed the ingredients approach and reported unit costs and resource use separately, a number comparable internationally [65]. Such an approach is vital for replication of the analyses in multiple settings or for example, across multiple health insurance funds within Austria, and also for international comparability and generalisability of the results.

Considering the sources of the applied unit costs, a number of studies relied on payer tariffs as valuation sources, a commonly adopted approach, also internationally [10, 30]. The inherent limitations of such an approach, i.e. that these estimates differ from opportunity costs, however, were discussed in a minority of six studies included in this review. This number seems low given the high share of studies that relied on payer tariffs across all cost components (Fig 3). Only one study [67] attempted to make cost-to-charge adjustments to the reimbursement data used for costing [68] by applying a multiplication factor to convert LKF reimbursement data (and ambulatory care physician tariffs) in order to approximate actual economic costs. Another study pointed out that inpatient costs as reflected in reimbursement data do not mirror actual resource consumption but instead represent the cost that society bears for these services [69]. For the Austrian ambulatory care sector, however, tariffs do not necessarily reflect true economic costs but are rather the result of political and business negotiation processes [24]. The same applies to the intramural sector in respect to using LKF values [24]. In Germany, where this trade-off between precision and pragmatism is also an issue, standard unit costs for selected services have been successfully calculated based on administrative data to reflect costs from a societal perspective [70]. In Austria, given the lack of such standardised unit costs, it seems especially surprising that not more than 15 studies applied micro-costing in their analyses.

While the above outlined methodological issues in the Austrian context might also be a reflection of historical practices and lacking guidance in general, they are nonetheless striking, especially given that most of the studies included in this review were published after the establishment of the relevant national guidance and several years after the publication of relevant international standards [10]. These methodological issues are, nonetheless, not necessarily unique to the Austrian setting [10] as suggested by several similar reviews from other countries [60, 65, 66, 71–80]. For example, an analysis of Australian economic evaluations published in 1995 found that an adequate description of cost measurement and valuation was only reported in 45% and 48% of all reviewed economic evaluations, respectively [71]. In a review of economic evaluations in the Spanish health care sector published in 2001, the authors found that the study perspective was explicitly stated only in 28% of all economic evaluations with 76% of the studies listing the sources of cost data comprehensively [72]. A review from Saudi Arabia found that 40% of the included economic evaluations reported the unit costs separately from the resource quantities used and only 10% clearly stated the year of cost data [65]. A systematic review of studies funded by the UK Health Technology Assessment Program also identified major discrepancies in resource costing methods including poorly defined study perspectives [80]. Another review conducted in 2014 on country-specific reviews of the quality of economic evaluations concluded that the reporting of costing methodology such as the source of the unit costs had not been adequately assessed, despite their crucial role in influencing economic evaluation results [25].

International examples point to two measures that have been implemented to overcome the aforementioned methodological issues in the costing process. Firstly, several countries including Canada, Australia and the Netherlands have published detailed costing guidelines. Secondly, selected countries (e.g. the UK [81–83], the Netherlands [4, 84–86] and Germany [5, 87, 88]) have also established standardised databases or lists of unit costs including (average) costs of the most commonly used health and social care services. In other countries, the systematic collection of other researchers’ unit costs (e.g. Farag et al. [89]) served as a starting point for such standardisation [90]. These measures could also help boost the credibility of economic evaluations for policy makers in Austria [62]. Indeed, the outlined comparison of GP costs and day surgery costs based on different sources and different costing methodologies revealed striking differences between estimates. In addition to better adherence to (detailed) guidelines, more homogenous costing methods and costing sources could tackle potential quality issues in future health economic analyses and hence increase the usability of such evidence also in the decision-making process.

### Limitations

The results of this review need to be interpreted in the context of its limitations. Despite extensive efforts to ensure inclusion of all eligible scientific articles and published reports, including a search of grey literature, contacting corresponding authors in case of unavailable full texts and extracting relevant referenced studies, studies could have been missed due to the limitations of systematic reviews or publication bias. Furthermore, although the quality assessment of costing methods and reporting was based on objective criteria by national and international guidelines (especially the allocation to the different types of valuation sources in Fig 3), it inevitably required some subjective judgments by the authors.

## Conclusion

The results of this review suggest considerable variability in the costing methods and their reporting standards in Austrian health economic analyses. Given the growing international concerns and actions about the need for better standardisation of costing methods [2] and the relevant observed inconsistencies in the Austrian context, updating and extension of the Austrian methodological guidelines seems to be necessary. Application of standardised unit costs for the most commonly used health and social care services as seen in other social insurance-based health care systems like Germany [5, 87, 88] and the Netherlands [4, 84–86] could further help increase the comparability and generalisability of health economic analyses in Austria as well [2]. Further potential inclusion of standardised inter-sectoral cost information would also encourage the incorporation of impacts outside the health care sector and promote inter-sectoral considerations [5, 91].

Based on this systematic literature review and extracted unit cost information, a publicly available and regularly updated Austrian online unit cost database has been set up by the Department of Health Economics, Center for Public Health at the Medical University of Vienna in February 2016 [92]. It contains all relevant unit costs identified in this review including their references and aims to serve as a starting point towards better harmonised costing methods in Austria. Works on a comprehensive, systematic, national-level reference cost library and the development of a detailed national costing guideline have also been initiated aiming to facilitate the uptake of relevant evidence in decision-making.

## Supporting information

S1 Text Search strategy: Embase and Medline. (DOCX)

S2 Text Grey literature search. (DOCX)

S1 Table PRISMA check list. (DOCX)
