# Supplementary material for: Costing evidence for health care decision-making in Austria: A systematic review
Source: PLoS One. 2017 Aug 14;12(8):e0183116. doi: 10.1371/journal.pone.0183116 (PMC5555669; doi:10.1371/journal.pone.0183116)
Supplement: S1 Text — (DOCX) [file pone.0183116.s001.docx]

**S1 Text. Search strategy: Embase and Medline.**

**Embase**

| 1. Health Economics/ |  |
| --- | --- |
| 2. exp Economic Evaluation/ |  |
| 3. exp Health Care Cost/ |  |
| 4. pharmacoeconomics/ |  |
| 5. 1 or 2 or 3 or 4 |  |
| 6. (econom$ or cost or costs or costly or costing or price or prices or pricing or pharmacoeconomic$).ti,ab. |  |
| 7. (expenditure$ not energy).ti,ab. |  |
| 8. (value adj2 money).ti,ab. |  |
| 9. budget$.ti,ab. |  |
| 10. 6 or 7 or 8 or 9 |  |
| 11. 5 or 10 |  |
| 12. letter.pt. |  |
| 13. editorial.pt. |  |
| 14. note.pt. |  |
| 15. 12 or 13 or 14 |  |
| 16. 11 not 15 |  |
| 17. (metabolic adj cost).ti,ab. |  |
| 18. ((energy or oxygen) adj cost).ti,ab. |  |
| 19. ((energy or oxygen) adj expenditure).ti,ab. |  |
| 20. 17 or 18 or 19 |  |
| 21. 16 not 20 |  |
| 22. animal/ |  |
| 23. exp animal experiment/ |  |
| 24. nonhuman/ |  |
| 25. (rat or rats or mouse or mice or hamster or hamsters or animal or animals or dog or dogs or cat or cats or bovine or sheep).ti,ab,sh. |  |
| 26. 22 or 23 or 24 or 25 |  |
| 27. exp human/ |  |
| 28. human experiment/ |  |
| 29. 27 or 28 |  |
| 30. 26 and 29 |  |
| 31. 26 not 30 |  |
| 32. 21 not 31 |  |
| 33. 0959-8146.is. |  |
| 34. (1469-493X or 1366-5278).is. |  |
| 35. 1756-1833.en. |  |
| 36. 33 or 34 or 35 |  |
| 37. 32 not 36 |  |
| 38. conference abstract.pt. |  |
| 39. 37 not 38 |  |
| 40. limit 39 to yr="2004 -Current" |  |
| 41. exp Austria/ |  |
| 42. "austri*".ti,pb,jn,sh,ab,kw,jx,ec,tw,fs. |  |
| 43. 41 or 42 |  |
| 44. 40 and 43 |  |

**Medline**

| 1. Economics/ |  |
| --- | --- |
| 2. exp "costs and cost analysis"/ |  |
| 3. Economics, Dental/ |  |
| 4. exp economics, hospital/ |  |
| 5. Economics, Medical/ |  |
| 6. Economics, Nursing/ |  |
| 7. Economics, Pharmaceutical/ |  |
| 8. (economic$ or cost or costs or costly or costing or price or prices or pricing or pharmacoeconomic$).ti,ab. |  |
| 9. (expenditure$ not energy).ti,ab. |  |
| 10. value for money.ti,ab. |  |
| 11. budget$.ti,ab. |  |
| 12. or/1-11 |  |
| 13. ((energy or oxygen) adj cost).ti,ab. |  |
| 14. (metabolic adj cost).ti,ab. |  |
| 15. ((energy or oxygen) adj expenditure).ti,ab. |  |
| 16. or/13-15 |  |
| 17. 12 not 16 |  |
| 18. letter.pt. |  |
| 19. editorial.pt. |  |
| 20. historical article.pt. |  |
| 21. or/18-20 |  |
| 22. 17 not 21 |  |
| 23. exp animals/ not human/ |  |
| 24. 22 not 23 |  |
| 25. bmj.jn. |  |
| 26. "cochrane database of systematic reviews".jn. |  |
| 27. health technology assessment winchester england.jn. |  |
| 28. or/25-27 |  |
| 29. 24 not 28 |  |
| 30. limit 29 to yr="2004-Current" |  |
| 31. austri* |  |
| 32. exp Austria/ |  |
| 33. "austri*".ab,hw,in,jn,jw,kw,ot,sh,ti,tw,xs. |  |
| 34. 32 or 33 |  |
| 35. 30 and 34 |  |
