# Supplementary material for: Costing evidence for health care decision-making in Austria: A systematic review
Source: PLoS One. 2017 Aug 14;12(8):e0183116. doi: 10.1371/journal.pone.0183116 (PMC5555669; doi:10.1371/journal.pone.0183116)
Supplement: S2 Text — (DOCX) [file pone.0183116.s002.docx]

**S2 Text. Grey literature search.**

For the grey literature search, key players were identified based on the Austrian HTA (Health Technology Assessment) guide, including the Department for Evidence-based Medicine and Clinical Epidemiology of Danube University Krems, Austrian Public Health Institute (Gesundheit Österreich GmbH), Main Association of Austrian Social Security Institutions – Evidence-based Economic Health Care (Hauptverband der österreichischen Sozialversicherungsträger – Evidenzbasierte wirtschaftliche Gesundheitsversorgung), Institut für Allgemeinmedizin und evidenzbasierte Versorgungsforschung, UMIT Institute of Public Health, Medical Decision Making and Health Technology Assessment, JOANNEUM RESEARCH Forschungsgesellschaft mbH, HEALTH – Institut für Biomedizin und Gesundheitswissenschaften, Ludwig Boltzmann Institute of Health Technology Assessment (Ludwig Boltzmann Institut für Health Technology Assessment). In addition, the Institute for Pharmaeconomic Research (Institut für Pharmaökonomische Forschung) and Main Association of Austrian Social Security Institutions – Health Economics were included. Hand searches for “Kosten*” and “cost*” on their websites were carried out and newly identified, relevant journal publications and project reports were included based on their title and abstract.
